# Supplementary material for: Polarity transitions of narrow bipolar events in thundercloud tops reaching the lower stratosphere
Source: Nat Commun. 2024 Aug 26;15:7344. doi: 10.1038/s41467-024-51705-y (PMC11347600; doi:10.1038/s41467-024-51705-y)
Supplement: Supplementary file 1 — Supplementary Information [file 41467_2024_51705_MOESM1_ESM.pdf]

Supplementary information

Polarity transitions of narrow bipolar events in thundercloud tops reaching the lower stratosphere

Feifan Liu[1,2,3], Torsten Neubert[3], Olivier Chanrion[3], Gaopeng Lu[1], Ting Wu[4], Fanchao Lyu[5], Weitao Lyu[5,6], Christoph Köhn[3], Dongshuai Li[3], Baoyou Zhu[1*] and Jiuhou Lei[1,7,8*]

1. CAS Key Laboratory of Geospace Environment, School of Earth and Space Sciences, University of Science and Technology of China, Hefei, China.
2. CMA-USTC Laboratory of Fengyun Remote Sensing, School of Earth and Space Sciences, University of Science and Technology of China, Hefei, China.
3. Department of Space and Earth Science and Technology, Technical University of Denmark (DTU Space), Kongens Lyngby, Denmark.
4. Department of Electrical, Electronic and Computer Engineering, Gifu University, Gifu, Japan.
5. Nanjing Joint Institute for Atmospheric Sciences, Nanjing, Jiangsu, China.
6. State Key Laboratory of Severe Weather, Chinese Academy of Meteorological Sciences, Beijing, China.
7. Mengcheng National Geophysical Observatory, University of Science and Technology of China, Hefei, China.
8. CAS Center for Excellence in Comparative Planetology, Hefei, China.

Corresponding author: Baoyou Zhu (zhuby@ustc.edu.cn) & Jiuhou Lei (leijh@ustc.edu.cn)

Supplementary Material:

•Supplementary Figures 1-8

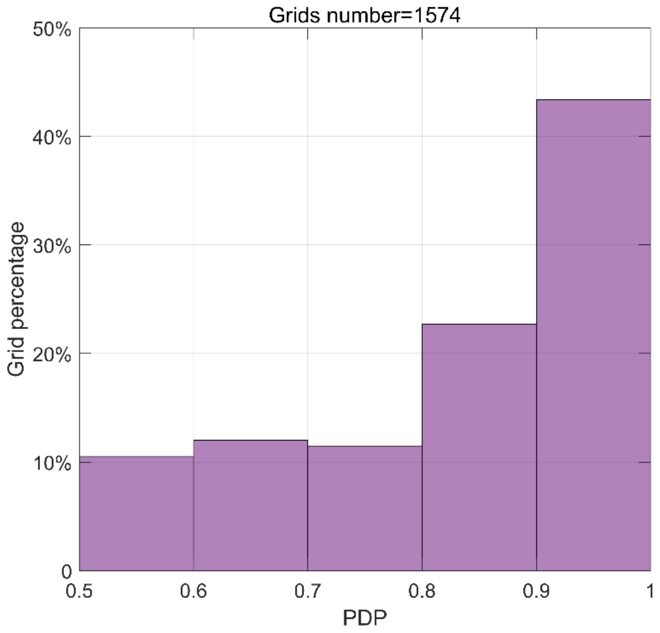

Supplementary Figure 1 | Histogram of the Proportion of the Dominant number of positive or negative Polarity to the total number (PDP) value in the grids with both polarities NBEs observed by the Guangzhou (GZ) station during one-year observation.

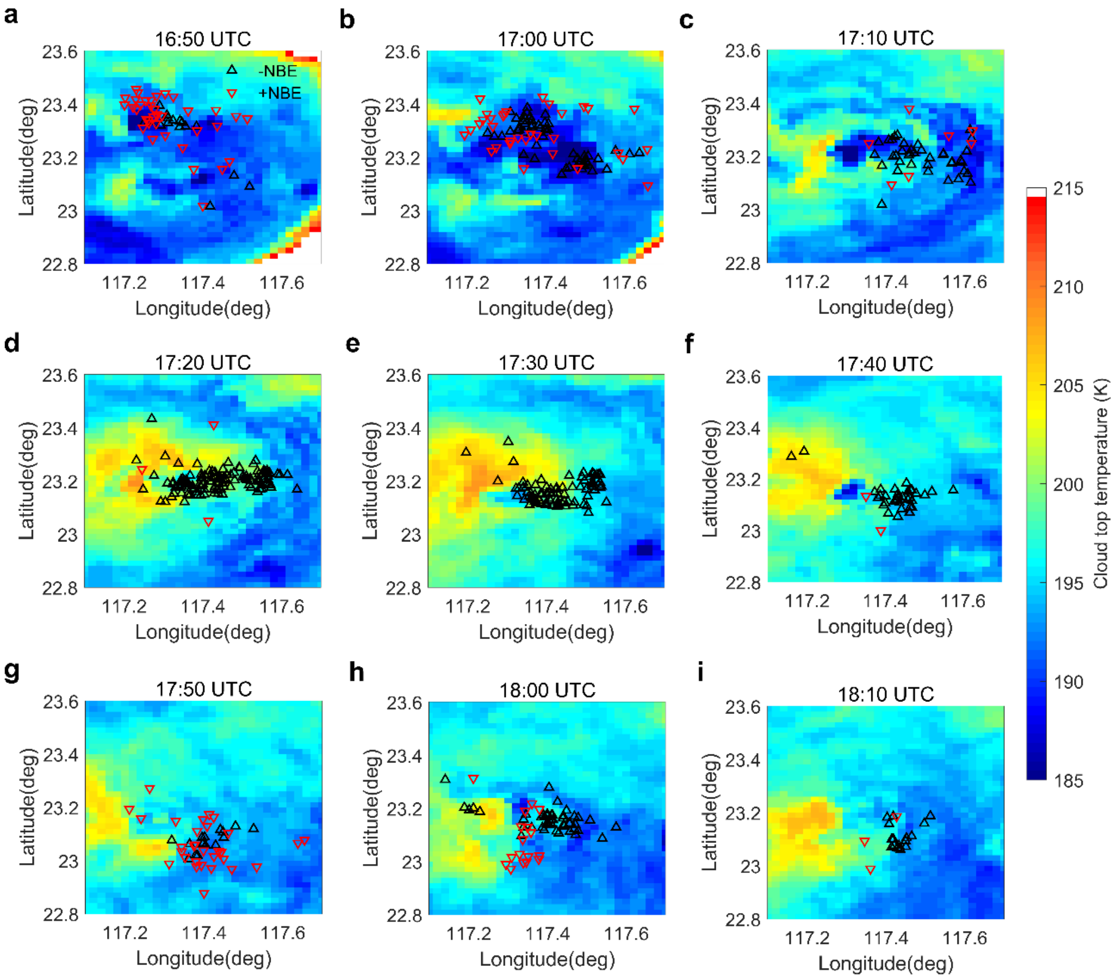

Supplementary Figure 2 | Positions of Narrow bipolar events (NBEs) overlaid on the cloud top temperature. a-i Evolution of both positive and negative NBE during 16:50-18:10 UTC. Black '△' represents negative NBEs and red '▽' presents positive NBEs. All NBEs within five minutes of the displayed time are shown.

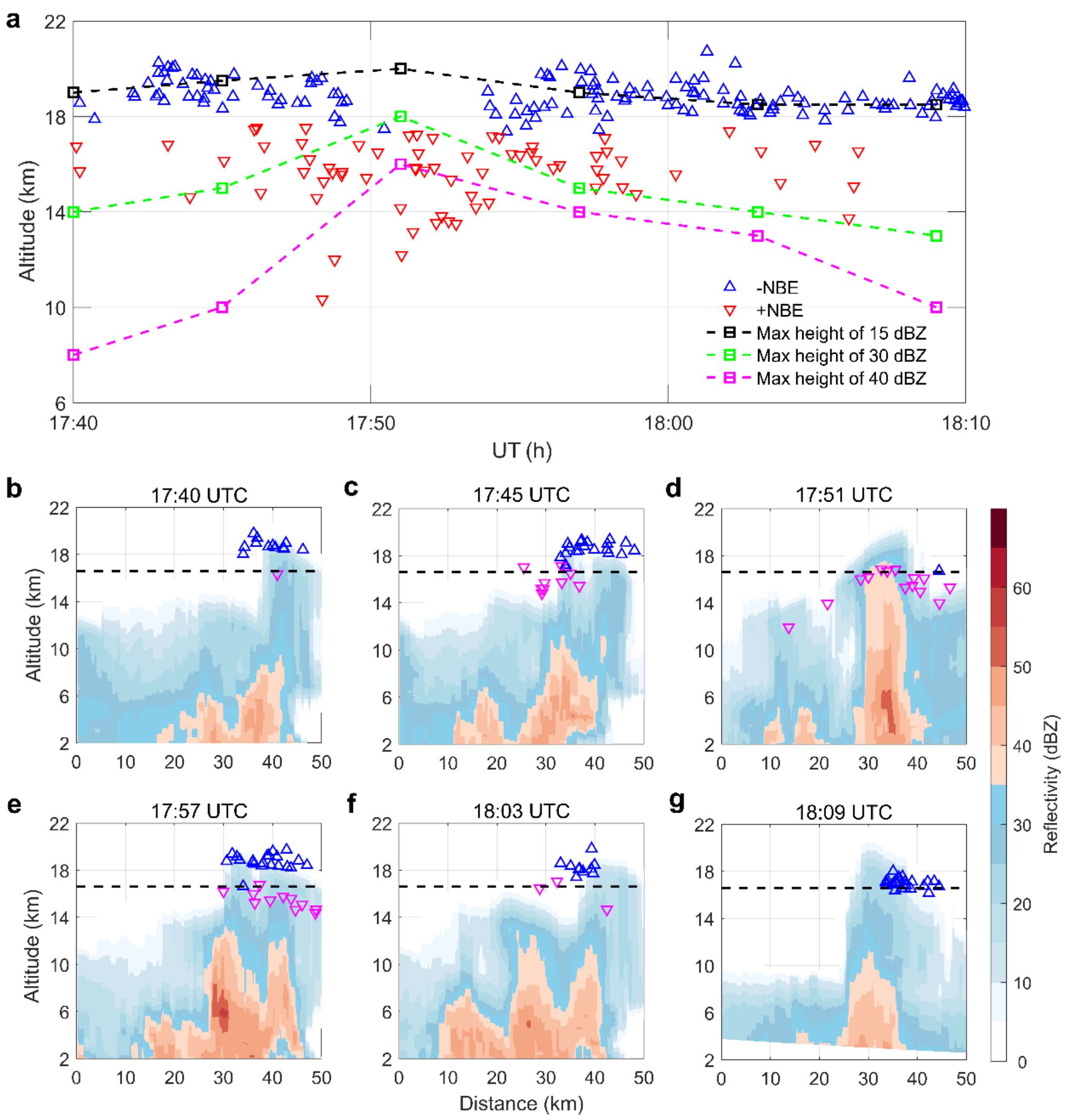

Supplementary Figure 3 | Vertical cross-section of radar along Narrow bipolar events (NBEs) outburst during 17:40-18:10 UTC. a Evolution of NBEs. The blue '△' presents negative NBEs while the red '▽' represents negative NBEs. The colored dashed lines represent the height variations of the reflectivity. The dashed black, green and pink lines present the maximum height of 15 dBZ, 30 dBZ and 40 dBZ derived from the vertical cross-section radar, respectively. **b-d** Vertical cross-section radar profile of NBEs during the upwelling stage. **e-g** Vertical cross-section radar profile of NBEs during the decaying stage. The deep red contour shows a large reflectivity echo (>35 dBZ); the dim blue contour indicated a small reflectivity echo (<35 dBZ). NBEs within 5 km off the cross-section line on the plane position indicator (PPI) are overlaid on the vertical cross-section. The dashed black line shows the tropopause height obtained from sounding data.

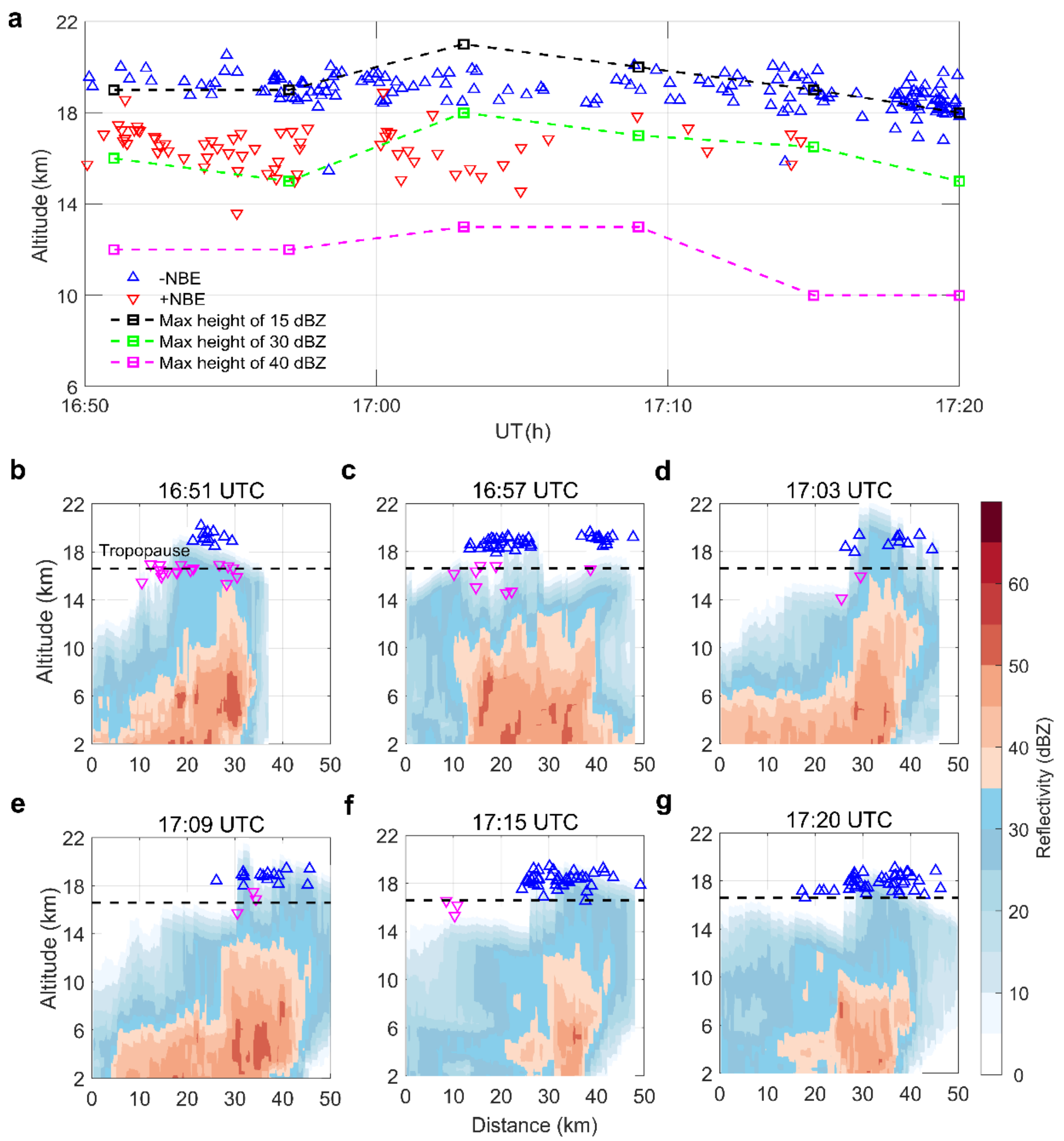

Supplementary Figure 4 | Vertical cross-section of radar alone Narrow bipolar events (NBEs) outburst during 16:50-17:20 UTC. a Evolution of NBEs. The color dashed line represents the height variations of reflectivity. **b-g** Vertical radar profile of NBEs. The deep red contour shows a large reflectivity echo (>35 dBZ); the dim blue contour indicated a small reflectivity echo (<35 dBZ). NBEs within 10 km off the cross-section line on the plane position indicator (PPI) are overlaid on the vertical cross-section. The dashed black line shows the tropopause height obtained from sounding data.

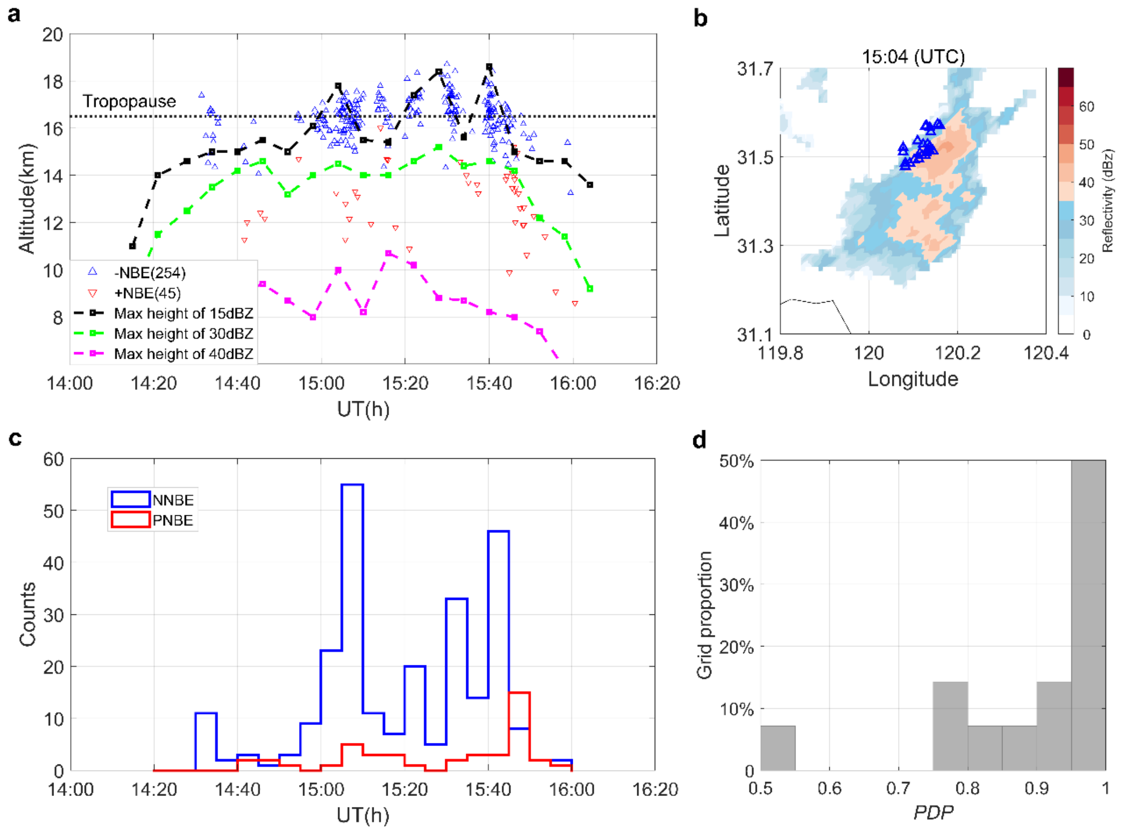

Supplementary Figure 5 | Detailed information of a supercell thunderstorm with the outburst of negative Narrow bipolar events (NBEs) on August 19, 2012. a Evolution of the NBEs and variations of the maximum height of radar reflectivity. **b** Negative NBEs overlapped on the radar reflectivity. **c** Time series of positive NBE (PNBE) and negative NBE (NNBE), respectively; the time bin size is 5 minutes. **d** Histogram of the Proportion of the Dominant number of positive or negative Polarity to the total number (*PDP*). The grid with the sum of NBEs larger than 5 and at least one negative NBE is counted. A total of 35 grids meets the criteria and are used to count the histogram of *PDP*.

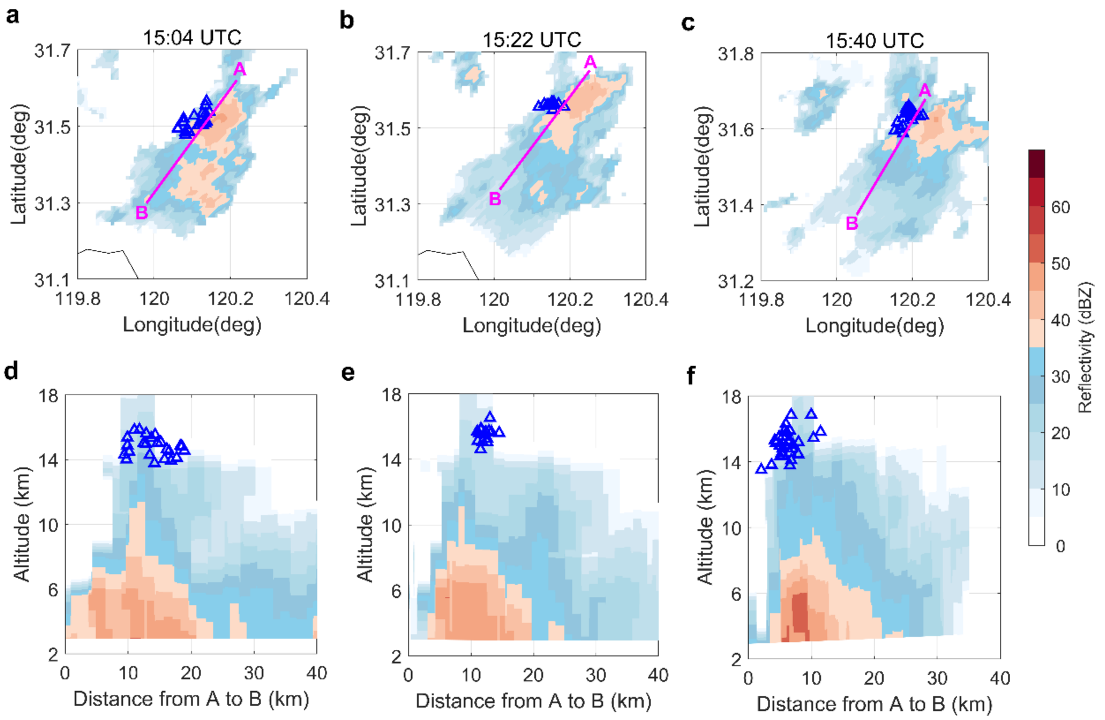

Supplementary Figure 6 | Vertical cross-section profile for the outbreak of negative Narrow bipolar events (NBEs) on August 19, 2012. a-c Negative NBEs overlapped on the radar reflectivity. **d-f** Vertical cross-section profile along line AB for the outbreak of negative NBEs. The blue '△' presents negative NBEs.

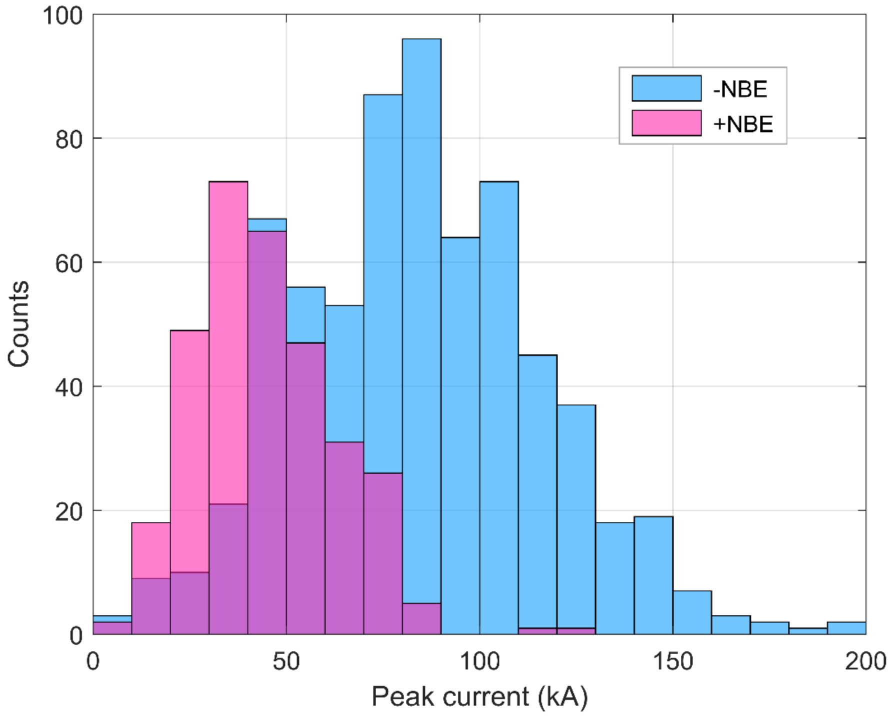

Supplementary Figure 7 | Peak current distributions of Narrow bipolar events (NBEs) in the tropical storm Haitang On 30 July 2017.

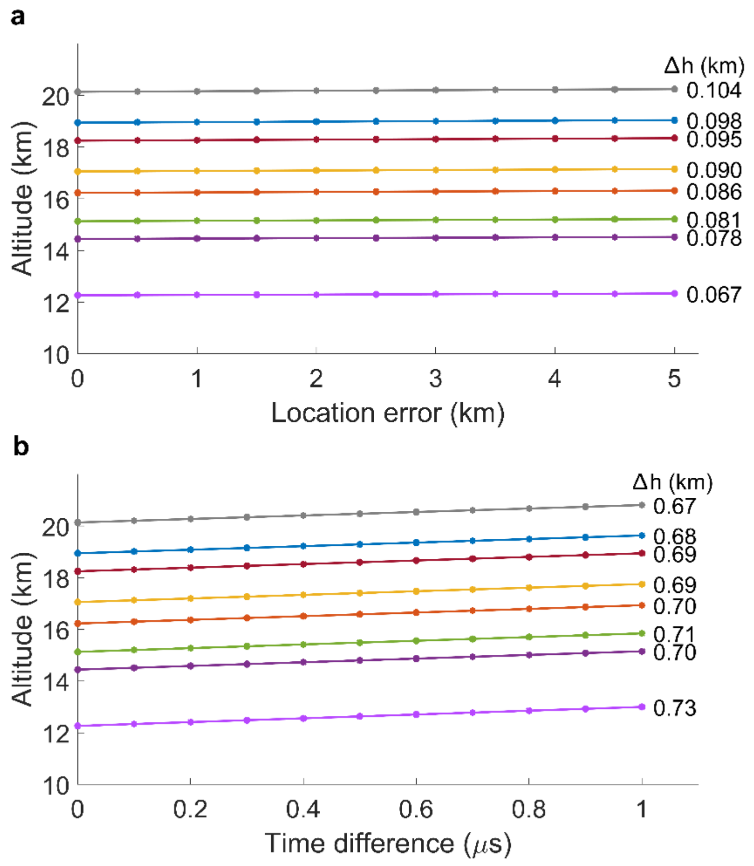

Supplementary Figure 8 | Variation of Narrow bipolar events (NBEs) altitudes with different location error and time error of reflected wave for nine NBEs ranging from 12-20 km. a-b The color line represents the estimated height of NBEs originating at different altitudes.
